# Relationship between Epicardial and Coronary Adipose Tissue and the Expression of Adiponectin, Leptin, and Interleukin 6 in Patients with Coronary Artery Disease

**DOI:** 10.3390/jpm12020129

**Published:** 2022-01-19

**Authors:** Olga V. Gruzdeva, Yulia A. Dyleva, Ekaterina V. Belik, Maxim Y. Sinitsky, Aleksandr N. Stasev, Aleksandr N. Kokov, Natalia K. Brel, Evgenia O. Krivkina, Evgenia E. Bychkova, Roman S. Tarasov, Olga L. Barbarash

**Affiliations:** Federal State Budgetary Scientific Institution “Research Institute for Complex Issues of Cardiovascular Diseases”, 650002 Kemerovo, Russia; o_gruzdeva@mail.ru (O.V.G.); sionina.ev@mail.ru (E.V.B.); sinitsky.maxim@gmail.com (M.Y.S.); stasan@kemcardio.ru (A.N.S.); kokoan@kemcardio.ru (A.N.K.); brel.n.k@mail.ru (N.K.B.); kriveo@kemcardio.ru (E.O.K.); eugenia.tarasowa@yandex.ru (E.E.B.); tarars@kemcardio.ru (R.S.T.); barb61@yandex.ru (O.L.B.)

**Keywords:** adiponectin, leptin, interleukin-6, epicardial adipose tissue, perivascular adipose tissue

## Abstract

Adipose tissue (AT) is an endocrine and paracrine organ that synthesizes biologically active adipocytokines, which affect inflammation, fibrosis, and atherogenesis. Epicardial and perivascular fat depots are of great interest to researchers, owing to their potential effects on the myocardium and blood vessels. The aim of the study was to assess the expression and secretion of adipocytokine genes in the AT of patients with coronary artery disease (CAD) and patients with aortic or mitral valve replacement. This study included 84 patients with CAD and 50 patients with aortic or mitral valve replacement. Adipocytes were isolated from subcutaneous, epicardial (EAT), and perivascular AT (PVAT), and were cultured for 24 h. EAT exhibited the lowest level of adiponectin gene expression and secretion, regardless of nosology, and high expression levels of the leptin gene and interleukin-6 (IL-6). However, EAT adipocytes in patients with CAD were characterized by more pronounced changes in comparison with the group with heart defects. High leptin and IL-6 levels resulted in increased pro-inflammatory activity, as observed in both EAT and PVAT adipocytes, especially in individuals with CAD. Therefore, our results revealed the pathogenetic significance of alterations in the adipokine and cytokine status of adipocytes of EAT and PVAT in patients with CAD.

## 1. Introduction

Atherosclerosis-related cardiovascular disease (CVD) remains one of the main causes of death in industrialized countries, despite significant achievements in modern medicine [1]. Moreover, the increasing incidence of obesity is likely to aggravate this problem. Adipose tissue (AT) is universally regarded as an endocrine and paracrine organ that synthesizes biologically active molecules. Adipocytokines are involved in various processes, including inflammation, fibrosis, and atherogenesis [2,3,4]. It has been shown that local fat depots, such as epicardial (EAT) and perivascular AT (PVAT), are associated with increased cardiovascular events, independent of traditional risk factors [5]. In addition, reports have shown differences in the matrix RNA (mRNA) expression of adipokines in epicardial and subcutaneous AT (SAT) [6]. As different fat depots are characterized by their unique adipocytokinome and secretome, studies on the role of AT adipocytokines at different locations and CVD are relevant.

EAT is of particular interest owing to its close location to the coronary arteries and its possible paracrine action on them [7]. When inflammatory processes are activated, the epicardium becomes a site of impaired adipogenesis, which leads to the secretion of pro-inflammatory adipokines and fibrosis of the atrial and ventricular myocardium. PVAT is also of interest, since its heterogeneity determines the functions of ATs-protective or atherogenic. EAT and PVAT have been shown to be associated with coronary artery disease (CAD) [8]. However, the mechanisms by which adipocytokines affect the pathogenesis of CVD are still not fully understood. Thus, it is necessary to study the expression of adipokine genes in AT in various sites to develop potential therapeutic interventions against pathological activation of AT in CVD.

## 2. Material and Methods

### 2.1. Study Inclusion Criteria

Inclusion criteria consisted of the following: CAD, presence of indications for coronary artery bypass grafting (CABG) (based on coronarography data), patient age up to 75 years, and patient consent for participating in the study. Exclusion criteria were as follows: patient age greater than 75 years; presence of type 1 and type 2 diabetes mellitus (DM) as evident in his/her medical history and/or revealed during the hospitalization examination, acute of pervious myocardial infarction (MI), presence of clinically significant comorbidities (anemia, kidney and liver failure, oncological and infectious–inflammatory diseases) in the period of exacerbation, autoimmune diseases, and refusal to participate in the study.

### 2.2. Study Population

The study included 125 patients with CAD and indications for direct myocardial revascularization by CABG. The study group consisted of 31 women (25%) and 94 men (75%), of an average age of 65.32 (57.35; 69.16) years (Table 1). In the patient history, hypertension, smoking, angina pectoris, and burdened heredity for cardiovascular pathology were recorded more often. In total, 86 patients had a previous history of MI, and 11 people had a history of stroke. The comparison group included 120 patients with heart defects (aortic or mitral valve replacement), consisting of individuals of an average age of 61.22 (55.49; 66.65) years (Table 1) without coronary atherosclerosis (as per coronary angiography). Individuals in group with heart defects were characterized by a low percentage of patients with cardiovascular risk factors.

All patients were administered standard antianginal and antiplatelet therapies throughout the observation period and in-hospital treatment period (Table 1).

### 2.3. Measurement of the Area of SAT, EAT, and PVAT

**Figure 1:**
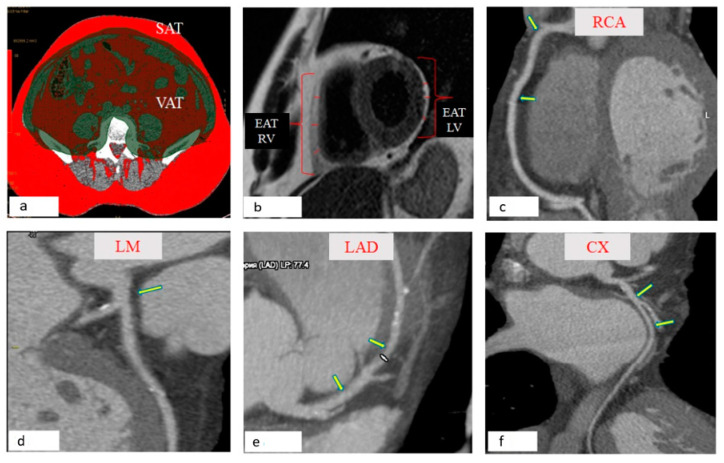
Morphometric assessment of local fat depots. (**a**) Slices centered at the L4–L5 disc spaces were selected. Visceral fat was defined as the fat enclosed by the visceral cavity. Subcutaneous fat was defined as fat outside the visceral cavity, not including that located within the muscular fascia. The total visceral and subcutaneous fat area (cm^2^) were automatically calculated using a workstation (Leonardo, Siemens, Germany). (**b**) EAT was defined as the adipose tissue located between the visceral layer of the pericardium and outer surface of the myocardium. EAT was measured on the horizontal long-axis plane in end-diastolic phase. The EAT thickness of the right and left ventricular free wall was recorded by averaging the measurements of 3 different levels. (**c**) The thickness of the PVAT right coronary artery (RCA) was measured at the level of the proximal third of the RCA, for which the orifice of the acute margin artery is the distal end, in the projection of the middle third of the RCA, bounded by the arteries of the acute margin and the posterior interventricular branch of the right coronary artery. (**d**) The thickness of the PVAT of the left coronary artery (LCA) was measured at the level of the left main (LM) coronary artery (Figure 1d). (**e**) The PVAT was assessed at the level of the proximal and middle third of the anterior descending artery (LAD). The proximal third of LAD is a site from the orifice to the origin of the first diagonal artery, while the middle third of the LAD is limited by the orifices of the first and second diagonal arteries. (**f**) The thickness of the PVAT was estimated at the level of the proximal third of the circumflex coronary arteries (CX), which is limited by the ostium of the CX and the branch of the obtuse edge and at the level of the middle third of the CX-a segment from the branch of the obtuse edge to the orifice of the posterior interventricular artery.

For all patients, the area was measured as abdominal AT, namely, its visceral and subcutaneous components, using multi-spiral computed tomography (MSCT) on a Siemens Somatom 64 computed tomography scanner (Siemens Healthcare, Erlangen, Germany). Scanning was performed at the level of vertebral bodies L4–L5 in the craniocaudal direction. At the level of the intervertebral disc L4–L5, the area of abdominal adipose tissue (AAT) was measured in a semi-automatic mode. Further, the area of visceral adipose tissue (VAT) was determined on the obtained image by highlighting the contour of the peritoneum. The area of SAT was calculated mathematically by subtracting the area of VAT from the area of AAT (Figure 1a).

The determination of EAT thickness was performed by magnetic resonance imaging (MRI) on an Exelart Atlas 1.5-T MR imager (Toshiba, Tokyo, Japan). Thickness measurements of EAT was performed on images oriented along the short axis of the heart. EAT thickness was measured at three points along the anterior wall of the right ventricle, after which the average value was calculated. In addition, the same method was used to measure EAT thickness at the back wall of the left ventricle, followed by the calculation of the mean value (Figure 1b).

Determination of the thickness of the PVAT at different anatomical locations was performed using the MSCT method with the following parameters: 1 mm axial slice thickness, an image matrix of 512 × 512, tube voltage of 120 kV, and a tube current of 100 mA. The MSCT images were acquired using bolus contrast, followed by a quantitative evaluation. The thickness of the PVAT of the right coronary artery (RCA) was measured at the level of the proximal third and in the projection of the middle third of the RCA (Figure 1c). The thickness of the PVAT of the left coronary artery (LCA) was measured at the level of the left main (LM) coronary artery (Figure 1d). The thickness of the PVAT of the anterior descending coronary artery (LAD) was measured at the level of the proximal and middle third of the LAD (Figure 1e). The thickness of the PVAT of the circumflex coronary arteries (CX) was measured at the level of the proximal third of the CX (Figure 1f). The obtained DICOM images were processed on a “Leonardo” multimodal workstation.

### 2.4. Cell Collection

#### 2.4.1. Obtaining Biopsies of AT from Various Sites

Biopsies of 3–5 g of SAT, EAT, and PVAT samples were obtained during coronary bypass surgery and heart defects (aortic or mitral valve replacement). SAT samples were obtained from the subcutaneous tissue of the lower angle of the mediastinal wound. EAT samples were obtained from the right heart (right atrium and right ventricle), and PVAT samples were obtained from the area of the right coronary artery. The AT samples were placed in Hanks’ Balanced Salt Solution (Merck KGaA, St. Louis, MO, USA) containing penicillin (100 U/L), streptomycin (100 mg/mL), and gentamicin (50 μg/mL).

**Figure 2:**
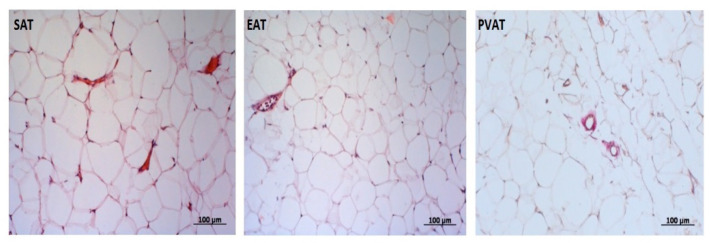
Representative microphotographs of hematoxylin and eosin (upper panels) stained samples of subcutaneous (SAT), epicardial (EAT) and perivascular (PVAT) from patients with coronary artery disease. Scale bar = 100 μm.

Adipose depots were harvested and fixed overnight in 10% neutral buffered formalin. Sections obtained from paraffin-embedded tissues were stained with hematoxylin and eosin (H&E) using standard protocols (Figure 2).

#### 2.4.2. Adipocyte Extraction and Culture

Adipocytes were isolated from ATs under sterile conditions in a laminar flow hood (BOV-001-AMS MZMO, Millerovo, Russia) using a previously described method [9]. Adipose tissue samples (3–5 g) were crushed to 1–3 mm^3^ fragments and incubated in a collagenase type I solution (0.5 mg/mL) (Thermo Fisher Scientific, Waltham, MA, USA) containing 200 nM adenosine (Merck KGaA, St. Louis, MO, USA) at 37 °C for 30 min. Then, the adipocytes were poured through a Falcon™ 100 μm sterile mesh (Thermo Fisher Scientific, Waltham, MA, USA) and washed with Gibco^®^ M199 culture medium (Thermo Fisher Scientific), 1% 4-(2-hydroxyethyl)-1-piperazineethanesulfonic acid buffer (Thermo Fisher Scientific), 1% l-glutamine with penicillin and streptomycin (Thermo Fisher Scientific), 0.4% amphotericin B (Thermo Fisher Scientific), glucose of 5 mmol/L, 10% fetal bovine serum (Thermo Fisher Scientific). The volume of the liquid containing the cells was adjusted to 5 mL using culture medium, and the cells were centrifuged for 2 min at 200× *g*. Isolated adipocytes (supernatant) were placed in a separate tube, and the volume was adjusted to 1 mL with culture medium. Adipocytes were counted in a Goryaev’s chamber. Cell viability was evaluated according to the method described by Suga et al. [10]. Adipocytes (20 × 10^5^) were seeded into a 24-well plate (Greiner Bio One International GmbH, Kremsmünster, Austria), and the volume in each well was adjusted to 1 mL with culture medium. The plates were incubated for 24 h at a temperature of 37 ± 1 °C in an atmosphere of 5% carbon dioxide and 10% oxygen. Subsequently, the adipocytes were carefully extracted from the surface of the culture medium to determine the expression of adipokine and cytokine genes and the culture medium was also extracted from the bottom of the wells for the determination of adipokine and cytokine levels by enzyme-linked immunoassay (ELISA).

### 2.5. Laboratory Assays

#### 2.5.1. RNA Extraction

Total RNA purification from isolated adipocytes was performed using the commercial RNeasy^®^ Plus Universal Mini Kit (Qiagen, Hilden, Germany), according to the manufacturer’s protocol with slight modifications, as described previously [11]. Extracted RNA was stored at −70 °C until use.

The quantity and quality of purified RNA were assessed using a NanoDrop 2000 Spectrophotometer (Thermo Fisher Scientific) by measuring the light absorbance at 280 nm, 260 nm, and 230 nm and calculating the 260/280 (A260/280) and 260/230 (A260/230) ratios. The integrity of the RNA was determined by electrophoresis in agarose gels, followed by visualization using the Gel Doc™ XR + System (Bio-Rad, Hercules, CA, USA).

#### 2.5.2. cDNA Synthesis

Single-stranded cDNA was synthesized using the High-Capacity cDNA Reverse Transcription Kit (Applied Biosystems, Foster City, CA, USA) on a VeritiTM 96-Well Thermal Cycler (Applied Biosystems, Waltham, MA, USA). Reverse transcription was performed using the program settings suggested by the kit’s manufacturer. The quantity and quality of synthesized cDNA were assessed using a NanoDrop 2000 Spectrophotometer. Samples were stored at −20 °C.

#### 2.5.3. Real Time Quantitative Polymerase Chain Reaction (qRT-PCR)

Expression of the adiponectin, leptin, leptin receptor and interleukin-6 (IL-6) genes was evaluated by quantitative real-time polymerase chain reaction (qPCR) using TaqManTM Gene Expression Assays (ADIPOQ Hs00605917_m1, LEP Hs00174877_m1, LEPR Hs00174497_m1, IL6 Hs00174131_m1, Applied Biosystems, Waltham, MA, USA) on a ViiA 7 Real-Time PCR System (Applied Biosystems, Waltham, MA, USA). For the negative control, we used 20 µL of the reaction mixture with no cDNA template. Three technical replicates were prepared for each sample and negative control. Results were normalized for three reference genes, *HPRT1* (hypoxanthine phosphoribosyltransferase-1), *GAPDH* (glyceraldehyde-3-phosphatedehydrogenase), and *B2M* (beta-2-microglobulin). To assess the effectiveness of PCR, amplification graphs and standard curves were analyzed using QuantStudioTM Real-Time PCR Software v.1.3 (Applied Biosystems). The expression of the studied genes (normalized quantification ratio, NRQ) was calculated by the Pfaffl method and is represented on a logarithmic (log10) scale as the fold change relative to control samples [12].

#### 2.5.4. ELISA

The concentration of adiponectin, leptin (Human Total Adiponectin/Acro30, DRP300; Human Leptin, DLP00) in the culture medium of adipocytes was determined by ELISA using test systems from R&D Systems, Inc. (Minneapolis, MN, USA) and IL-6 was determined using the test system Interleukin-6 BMS213-2 from eBioscience (Vienna, Austria).

### 2.6. Statistical Analysis

Statistical analysis was performed using GraphPad Prism 6 (GraphPad Software, La Jolla, CA, USA) and Statistica software, 10.0 (Dell Software, Inc., Round Rock, TX, USA). The Kolmogorov–Smirnov test was used to verify the normal distribution of data. The data were not normally distributed. Therefore, nonparametric methods were used. Data were presented as median (Me) and 25th and 75th quartiles (Q1; Q3). Two independent groups were compared using the Mann–Whitney U test. Three independent groups were compared using the Kruskal–Wallis test by ranks, followed by a pairwise comparison using the nonparametric Mann–Whitney test with the Bonferroni correction. Categorical variables are expressed as percentages and compared using chi-squared test or Fisher’s exact test. A *p*-value of less than 0.05 was considered statistically significant.

To identify the relationship between quantitative indicators and a qualitative trait, the method of stepwise logistic regression analysis and ROC analysis with the construction of the characteristic ROC curve and the calculation of the area under the ROC curve were used. The area under the ROC curve exceeding 0.70 was considered diagnostically significant. Using ROC analysis, the threshold values of the morphometric characteristics of AT of various localization were established.

## 3. Results

### 3.1. Visualization of Local Fat Depots (Epicardial, Perivascular, Abdominal) in Patients with Coronary Heart Disease

We observed that the thickness of LV EAT and RV EAT was, on average, 1.2 times higher in patients with CAD than in the group of patients with heart defects (Table 2).

In a comparative analysis of the quantitative parameters of PVAT in the study groups of patients with CAD, we observed that the thickness of PVAT of the proximal and middle third of the RCA was higher by 60% and 75%, respectively, than in patients with heart defects. In patients with CAD, the thickness of the PVAT at the level of the trunk of the left coronary artery (LCA) and the thickness at the level of the proximal and middle third of the RCA were 24%, 30%, and 31% higher, respectively, in comparison with that of group patients with heart defects. There was also an increase in the thickness of the PVAT at the level of the proximal and middle third of the circumflex artery in patients with CAD by 21% and 30%, respectively, than that of group patients with heart defects. Upon assessing the abdominal fat depot in patients with CAD, we observed that the VAT area exceeded the parameters of patients with heart defects by 1.2 times (Table 2).

The next step was to identify the relationship between the morphometric parameters of AT and the presence of atherosclerosis in the coronary arteries using logistic regression. The results are presented in Table 3. We observed that the parameter with the closest association with the presence of atherosclerosis in the coronary arteries is the thickness of the EAT LV, the thickness of the PVAT of the LCA trunk, the proximal third of the anterior descending artery, the proximal third of the circumflex artery, and the area of the VAT.

Threshold values were set for quantitative indicators of fat storage most associated with coronary atherosclerosis. Thus, for the thickness of the EAT LV, this parameter was 3.3 mm (sensitivity = 87.4, specificity = 88.9) for the thickness of the PVAT LCA was 3.4 mm (sensitivity = 86.4, specificity = 87.2), *p*/3 anterior descending artery was 3.7 mm (sensitivity = 88.3, specificity = 87.5), and *p*/3 circumflex artery was 3.35 mm (sensitivity = 86.4, specificity = 88.1)). The critical level of the VAT area was 106.1 cm^2^ (sensitivity = 90.9, specificity = 89.2).

### 3.2. Adiponectin Gene Expression and Concentration of Adiponectin in the Daily Adipocyte Culture of Various Fat Depots

**Figure 3:**
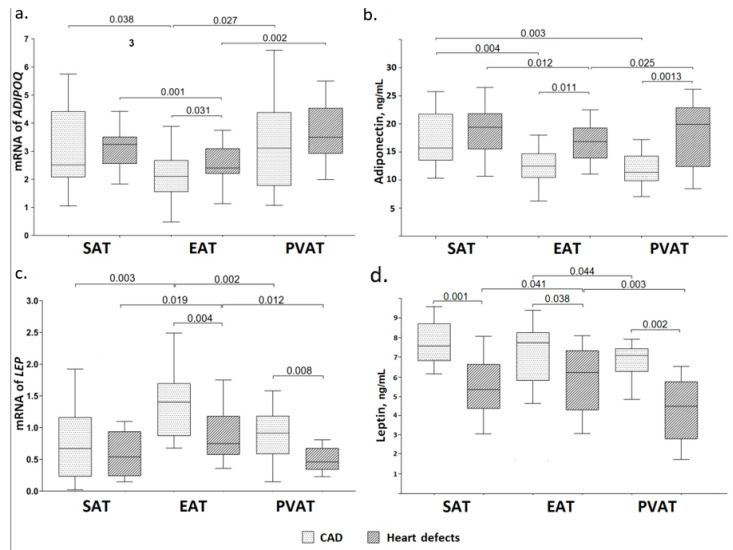
Adipokine gene expression and secretion in various fat depots in the daily culture of adipocytes derived from patients with CAD and patients with heart defects (aortic or mitral valve replacement). (**a**) Adiponectin gene expression (**b**) Adiponectin concentration (**c**) Leptin gene expression (**d**) Leptin concentration Notes: *ADIPOQ*-gene of adiponectin, *LEP*-gene of leptin, CAD-patients with coronary artery disease and those with indications of direct myocardial revascularization by coronary artery bypass grafting, Heart defects patients with aortic or mitral valve replacement, *p*-significant differences as compared to subcutaneous adipocytes, (*p* ≤ 0.05).

Adipocytes in the EAT expressed the lowest amount of *ADIPOQ* relative to adipocytes in different locations in both groups (Figure 3a).

In patients with CAD, *ADIPOQ* expression in the EAT was significantly lower than in the culture of SAT and PVAT (1.2 times (*p* = 0.038) and 1.5 times (*p* = 0.027), respectively). Similarly, in group patients with aortic or mitral valve replacement, *ADIPOQ* expression in EAT adipocytes was lower compared to adipocytes in the SAT and PVAT (1.4 times (*p* = 0.001) and 1.5 times (*p* = 0.002), respectively). Moreover, in Group 2, the mRNA level of *ADIPOQ* in EAT adipocytes was higher than that of patients with CAD by 1.2 times (*p* = 0.031). High mRNA levels of *ADIPOQ* were observed in the culture of PVAT. Further, *ADIPOQ* expression in cultures of SAT and PVAT adipocytes was not statistically different between patients with CAD and heart defects (Figure 1).

The concentration of adiponectin in the daily culture of EAT adipocytes was lower than that of SAT in both groups (1.3 times (*p* = 0.004) and 1.13 times (*p* = 0.012), respectively) (Figure 3b).

Moreover, adiponectin levels in group with heart defects were 1.4 times higher than in group patients with CAD individuals (*p* = 0.011). The lowest level of adiponectin secretion was detected in PVAT adipocytes of patients with CAD than that of adipocyte cultures at different locations. By contrast, in patients heart defects, adiponectin levels in the PVAT exceeded that of fat depots at different locations by 1.8 times (*p* = 0.001). Adiponectin concentrations in cultures of SAT adipocytes were not statistically different between groups patients with CAD and heart defects (Figure 3b).

### 3.3. Leptin Gene Expression and Concentration of Leptin in the Daily Adipocyte Culture of Various Fat Depots

High levels of *LEP* expression were observed in the culture of EAT adipocytes in patients of both groups compared with adipocytes at a different location (Figure 3c). In group patients with CAD, *LEP* expression was 1.9 times higher than in group with heart defects (*p* = 0.004). Thus, mRNA levels of the *LEP* gene in EAT adipocytes exceeded that of the SAT and PVAT adipocytes by 2.1 (*p* = 0.003) and 1.5 times (*p* = 0.002), respectively, among patients with CAD, and by 1.4 (*p* = 0.019) and 1.6 times (*p* = 0.012), respectively, in patients with heart defects. Moreover, in group patients with heart defects, the lowest mRNA level of the *LEP* was detected in the PVAT culture, which was two times (*p* = 0.008) lower than that in patients with CAD. However, *LEP* expression in SAT adipocyte cultures did not show a statistically significant difference, independent of nosology (Figure 3c).

Leptin concentration was also the highest in the culture of EAT adipocytes in both groups, whereas among patients with CAD, it exceeded that group of heart defects by 1.2 times (*p* = 0.038) (Figure 3d). However, statistically significant differences compared with SAT were only found in individuals of patients with heart defects (1.2 times, *p* = 0.041). The lowest leptin concentration was observed in PVAT adipocytes compared to adipocyte cultures of other locations, and statistically significant differences were found relative to EAT adipocytes. Leptin levels in the PVAT were 1.1 times (*p* = 0.044) lower than that of the EAT in group patients with CAD and 1.5 times (*p* = 0.003) lower than that of group with heart defects. Further, leptin concentration in the PVAT adipocyte supernatant of patients with CAD was 1.5 times higher than that of comparison group (*p* = 0.002). There were no statistically significant differences in leptin concentration in the culture of SAT adipocytes between groups (Figure 3d).

### 3.4. IL-6 Gene Expression and Concentration of IL-6 in the Daily Adipocyte Culture of Various Fat Depots

Upon measuring pro-inflammatory *IL6* expression in a 24 h culture of adipocytes at various locations, maximum levels were observed in the EAT relative to the SAT and PVAT in group patients with CAD (2.1 times with *p* = 0.0012 and 1.4 times with *p* = 0.0024, respectively) and comparison group relative to the PVAT (1.9 times with *p* = 0.0002). Moreover, mRNA level of the *IL6* in the EAT of patients with CAD exceeded that of group patients with heart defects by 1.4 times (*p* = 0.0013) (Table 4). Further, *IL6* expression in PVAT adipocytes in group patients with CAD was 1.9 times (*p* = 0.0001) higher than that of patients with heart defects. Subcutaneous adipocytes in both groups did not show differences in IL-6 gene expression.

The concentration of IL-6 in the EAT adipocyte culture was higher compared to SFU and PVAT, regardless of nosology (Table 4). Thus, in patients with CAD, the concentration of IL-6 in the EAT was 2.5 times and 1.8 times higher than that in the SAT and PVAT, respectively. In Group 2, the EAT was 1.5 times and 1.8 times higher than that in the SAT and PVAT, respectively. Moreover, the concentration of IL-6 in the EAT and PVAT in patients with CAD exceeded the level of patients with heart defects by 1.4 times (*p* = 0.022 and *p* = 0.018, respectively). IL-6 secretion in SAT adipocytes did not differ between groups.

### 3.5. Relationship between Gene Expression and Secretion of Adipocytokines in AT with Indicators of Morphometric Characteristics of Local Fat Depots of the Heart and Blood Vessels in Patients with CAD

Patients with CAD were divided into groups in accordance with the threshold values of the size of fat depots to identify the factors most associated with the expression and secretion of adipocytokines and morphometric characteristics of AT of ectopic localization. For example, the number of patients in whom the values of the thickness of the EAT LV, PVAT LCA, *p*/3 anterior descending artery, *p*/3 circumflex artery, and the area of the VAT exceeded the threshold values amounted to 60 (48%).

Among all the studied indicators of adipocytokine status, the expression of *ADIPOQ* in the EAT and PVAT, *LEP* in the EAT, *IL6* in the EAT and PVAT, the concentration of adiponectin in the EAT, and leptin in the EAT and PVAT were most associated with an increase in the size of local fat depots (Table 5).

## 4. Discussion

Recently, AT of the heart and blood vessels has been gaining attention as a new modifiable risk factor for the development and progression of CVD [13,14]. At the same time, it is well known that AT performs a number of key functions necessary for maintaining tissue and cellular myocardial homeostasis [15]. The dualism of the physiological and pathological effects of AT a given site is not fully understood, although it has been noted that an unfavorable course of CVD is associated with AT dysfunction in the heart and blood vessels. Further, fat deposits around the heart and blood vessels can play an important role in the pathogenesis of CVD owing to their anatomical proximity to vascular structures and the myocardium. It has been proven that the paracrine effect of PVAT on the vascular wall consists of the secretion of adipocytokines involved in vasoconstriction and vasodilation [16]. Moreover, adipocytes secrete adipokines and proinflammatory cytokines’ the level of the secretion is known to correlate with obesity [17].

One of the objectives of this study was a comparative assessment of the morphometric parameters of EAT in patients with coronary and non-coronary heart disease. The results obtained indicate that patients with CAD exhibited higher values of EAT thickness along the anterior wall of the RV, which indicates an uneven distribution of EAT along the heart surfaces. These features are presumably anatomically and physiologically determined. It has previously been shown that the left and right ventricles contain the same absolute amount of fat on their epicardial surfaces [18]. However, taking into account the obvious differences in the mass of the myocardium of the respective ventricles, the proportion of AT in the left ventricle was less than in the right one. At the same time, the maximum EAT thickness was observed in patients with CAD. According to Iantorno et al., an increase in the linear indicators of EAT was observed in patients with CAD and immunodeficiency in comparison with persons with no signs of CAD [(13.9 ± 3.1 mm) and (10.7 ± 3.3) mm, respectively (*p* = 0.001)] [19]. Similar results were obtained by Picard et al., who reported that the thickness of EAT in patients with CAD was 2.74 ± 2.4 mm, whereas it was (2.08 ± 2.1) mm (*p* = 0.0001) in healthy individuals [20]. An increase in the size of EAT in patients with CAD may be due to a low level of AT oxygenation due to impaired blood supply to the myocardium in coronary heart disease (AT of the heart is in general circulation with the myocardium). Previous studies have found an inverse relationship between adipocyte size and AT blood flow [21].

Statin therapy plays an important role in the primary and secondary prevention of cardiovascular disease. In addition to inhibiting β-hydroxy β-methylglutaryl-CoA reductase, statins can interfere with various processes such as signaling, differentiation, and cell proliferation. The modulation of EAT by statins may be one of the most important pleiotropic effects of this class of drugs. One study has also suggested a potential anti-inflammatory effect of statins on VAT, [22]. However, there is little evidence of an effect on VAT in the cardiac region.

Indeed, there are positive results from studies conducted for different durations. In the study by Alexopoulos, statin therapy for 12 months resulted in a decrease in EAT compared with placebo [23]. In a relatively recent study, intensive statin therapy for patients with CAD when used for 3 to 72 months also indicates a direct effect on EAT thickness (assessed by ECHO-CT) and inflammatory status [24].

In the experimental study by Ishihara, treatment of 3T3-L1 preadipocytes with different concentrations of pitavastatin and the daily administration of the drug to female mice showed that statins significantly reduce (by 16.8%) the number of hypertrophied adipocytes, the total mass of which remained unchanged [25]. Later, in another pilot study, it was similarly demonstrated that low concentrations of statins (10 mg/kg atorvastatin and 3 mg/kg rosuvastatin for 48 days) consistently reduce AT mass and adipocyte size in mice on a high-fat diet [26].

Thus, statins are undoubtedly potential drugs capable of directly affecting the AT of the heart and modulating the functional activity and size of adipocytes; however, for the manifestation of these effects, their long-term use from several months to 1 year is required. In our study, patients took statins for 5 days before CABG and, therefore, before receiving VT biopsies. Additionally, the measurement of the morphometric parameters of fat depots was carried out in the period from 1 to 4 days before the operation; therefore, we assume that statin therapy in our case could not significantly affect the results of the study.

This study also showed that there was an increase in the thickness of the PVAT at the level of the proximal and middle third of the RCA, the proximal and middle thirds of the LAD and CX, as well as the trunk of the LCA in patients with CAD, compared with patients with heart defects. Thus, there is a significant predominance of PVAT in the basin of the RCA. The data obtained are consistent with the results of the study by Demircelk et al. The average thickness of the PVAT was significantly higher in patients with signs of obstructive atherosclerotic lesions in comparison with those with no signs of CAD and minor signs of coronary atherosclerosis [27].

An increase in the PVAT indices in patients with CAD may be due to mechanisms similar to that operating in the EAT. A decrease in oxygen supply against the background of atherosclerotic lesions of the coronary arteries can affect the pericoronary AT, causing hypoxia, and leading to an increase in the size of adipocytes. In addition, studies conducted in recent decades have shown the participation of adventitia and the surrounding PVAT in the development of atherosclerosis in the coronary arteries [28].

The identified associations may be associated with an increase in the secretory activity of cardiac and vascular AT in patients with CAD. Previously, it was shown that the adipocytes of EAT and PVAT are characterized by impaired differentiation and disproportionate adipocytokine secretion, manifested by a decrease in adiponectin levels and an increase in leptin and IL-6 levels in comparison with adipocytes of SAT and perirenal adipocytes [29]. According to our results, adiponectin gene expression levels in the culture of EAT adipocytes was the lowest in patients of both groups. In addition, the level of adiponectin in the EAT and PVAT is associated with an increase in the size of local fat depots around the heart and blood vessels. The data obtained in our study is consistent with the study of Bambacea et al. [6]. and Iacobellis at al [30]. In a study by Eiras at al., a reduced mRNA level of the *ADIPOQ* detected in the EAT relative to the SAT in patients without CAD [31]. However, other authors have shown no differences between patients with CAD and patients with aortic or mitral valve replacement. For example, Iglesias et al. showed that adiponectin gene expression in the EAT was lower than in the SAT, but there were no statistically significant differences between patients after CABG and with valve replacement [32]. The authors explain this by gender characteristics, as mRNA level of the *ADIPOQ* in the EAT were higher in women. PVAT is also actively involved in atherogenesis, and it synthesizes proinflammatory molecules that are involved in the formation of unstable plaques that are prone to rupture and atherothrombosis and promote the progression of CAD [8,33]. Due to this close interaction, the PVAT is the first fat depot that recognizes and reacts to emerging changes in homeostasis through the synthesis and production of adipocytokines [34]. Adiponectin plays a crucial role, as its protective and anti-inflammatory properties are known. Our results demonstrate that PVAT had the highest level of adiponectin gene expression. Moreover, adiponectin was higher in group patients with heart defects. Similar data were demonstrated by Cybularz et al. [35]. Reduced adiponectin gene expression in combination with high expression of the leptin gene may be associated with the activation of key atherogenic pathways, contributing to the progression of CAD [36].

Based on the literature, it can be assumed that the levels of adipocytokine mRNA in the AT are not always a complete reflection of their concentration [37]. In this study, PVAT adipocytes of patients with CAD were characterized by the highest level of adiponectin gene expression, yet they exhibited the lowest concentration in the culture supernatants compared to adipocyte cultures at other locations. One of the reasons for this difference may be the low expression of adiponectin receptors in these fat depots. In a study by Guo at al., which assessed the role of adiponectin and its receptors in vivo, the authors found that angiotensin II (Ang II)-induced hypertension led to a significant decrease in the expression of both adiponectin and its AdipoR1 and AdipoR2 receptors in perivascular adipocytes and vascular cells [38]. Nacci et al. investigated the effect of infliximab on adiponectin gene expression in PVAT and showed a decrease in the level of adiponectin mRNA, AdipoR1, and AdipoR2 in mice with type 1 DM. Taken together, the findings suggest that the PVAT is a site of adiponectin/AdipoR dysregulation, and secondly, it is most susceptible to proinflammatory signals [39].

Another reason may be the long-term processes of post-translational modification and oligomerization of adiponectin. The observed high level of adiponectin in PVAT is due to the longer processes of “maturation” of adiponectin in CAD, which occur in the endoplasmic reticulum (ER) of adipocytes and are controlled by special proteins/chaperones in the ER such as Ero1-Lα. It is assumed that part of the synthesized adiponectin undergoes decay or proteolysis, and the delayed release of adiponectin is due to disruption of protein-chaperones that regulate adiponectin secretion from the cell [40]. In addition, Bauchea et al. showed that adiponectin was to be able to suppress its own production and expression of its AdipoR2 receptor in transgenic mice [41]. Further, Kadowaki et al. demonstrated a decrease in the number of adiponectin receptors in metabolic syndrome and obesity [42].

Leptin, the most studied adipokine marker of obesity, participates in the regulation of atherogenesis, thrombogenesis, and vascular revascularization. Further, it stimulates inflammatory processes, oxidative stress, and vascular smooth muscle cell hypertrophy. Leptin plays a role in the pathogenesis of hypertension, CAD, and type 2 DM and the development of their complications [43]. According to our data, a higher expression of the leptin gene was observed in the culture of EAT adipocytes compared to the SAT in patients. Furthermore, in patients with CAD, *LEP* expression was the highest in PVAT adipocytes. An increase in the level of *LEP* expression in EFT is associated with an increase in the size of fat depots in the heart and around the coronary vessels. This data is consistent with the data provided by other groups [44]. Polyakova et al. showed that in men, regardless of the presence of coronary heart disease, the *LEP* expression in the EAT was significantly higher than the SAT [45]. Furthermore, the authors explain the observed differences by the predominance of men among patients with CAD, and the incidence of more severe atherosclerotic lesions of the coronary arteries in male patients [46]. However, Iglesias et al. showed that *LEP* expression in the EAT was lower than the SAT from 46 patients who were undergoing heart surgery, coronary artery bypass surgery, or aortic or mitral valve replacement. Moreover, leptin mRNA expression in EAT was higher in women than men [32]. Increased leptin gene expression in the EAT culture of patients can have a negative effect on both adipocytes and cardiomyocytes by activating inflammatory and atherosclerotic processes [47].

Thus, the “protective” potential of AT depends on its localization. EFT adipocytes of CAD patients are characterized by changes in the adipocytokine system: low level of adiponectin gene expression against a high level of leptin gene expression and secretion compared to adipocytes at other locations.

IL-6 has been shown to produce various types of cells. However, adipocytes are also able to secrete proinflammatory IL-6 [48]. Approximately 30% of the IL-6 in the body is synthesized by AT, and its production is increased in overweight individuals. IL-6 is involved in the regulation of the energy balance of fat and muscle tissues, triggering regenerative mechanisms of immune protection and regulation of fat intake into AT. In addition, IL-6 is able to limit the inflammatory response by inhibiting the synthesis of a number of proinflammatory cytokines, including tumor necrosis factor-α (TNF-α) [49]. In vitro studies have shown that isolated visceral fat synthesizes more IL-6 than subcutaneous fat, and increased IL-6 production has been observed in adipocyte hypertrophy. plasma levels of IL-6 correlate with the area of epicardial and abdominal visceral AT in patients with coronary artery atherosclerosis, suggesting a potential effect of these fat depots on the development of atherosclerosis through paracrine rather than systemic effects [14].

We found that *IL6* expression in the daily culture of EAT adipocytes was the highest in comparison to the SAT and PVAT in both patients with CAD and those with aortic or mitral valve replacement. Moreover, the level of *IL6* mRNA in the EAT and PVAT in individuals with CAD was higher than in patients with heart defects. These identified features are consistent other studies. Shibasaki at al. found that *IL6* expression in the EAT was significantly higher than that in the SAT [50]. Moreover, *IL6* and *LEP* expression in the EAT was higher in patients with CAD, and *ADIPOQ* expression were comparable in both groups. In the SAT, *IL6* and *LEP* expression levels were moderately higher in patients with CAD compared to those without CAD. However, there were no differences in plasma cytokine levels between the two groups. [50].

Increased *IL6* expression and secretion in EAT and PVAT adipocytes can contribute to the high level of *LEP* expression and content observed in these types of AT. It is known that leptin has a stimulating effect on immune cells and is able to regulate the production of pro- and anti-inflammatory cytokines. [51]. In addition, some studies have noted homology in the structure of leptin and IL-6 receptors, also suggesting an interaction between leptin and circulating factors, through which cytokines inhibit binding of the leptin receptor to block its signaling activity in cell culture [52].

Experimental in vitro findings demonstrated that secretory products of adipocytes increase the secretion of inflammatory cytokines by macrophages and other immune cells, and pro-inflammatory cytokines, in particular IL-6, increase the transcription of leptin, which was confirmed by in vivo studies. Pro-inflammatory stimuli such as TNF-α and IL-6 itself can enhance IL-6 production in vitro. The content of IL-6 in AT is hundred times higher than in the plasma, suggesting important auto and paracrine regulatory functions in this tissue [53].

## 5. Conclusions

In conclusion, increased production of leptin and IL-6 and decreased production of cardioprotective adiponectin led to activation of immune cells and inflammation. This manifested primarily in EAT and PVAT adipocytes, especially in individuals with CAD, which creates favorable conditions for the development and progression of atherosclerosis. Moreover, leptin is able to affect the vascular wall and activate platelet aggregation, increasing the risk of thrombosis. Established changes in the content of the studied adipocytokines in epicardial adipocytes is a prognostically unfavorable sign in this category of patients. Therefore, the study of EAT revealed the pathogenetic significance of changes in the adipokine and cytokine status of adipocytes in patients with CAD.

## Figures and Tables

**Table 1:** Clinical and anamnestic characteristics of patients with coronary artery disease and heart defects.

Parameter	CAD (*n* = 125)	Heart Defects (*n* = 120)	*p*
Men, *n* (%)	97 (77.6)	60 (50)	0.011
Body mass index, kg/m^2^	28.89 (26.64; 32.12)	26.78 (23.21; 29.01)	0.069
Arterial hypertension, *n* (%)	118 (94.4)	32 (26.7)	0.001
Hypercholesterolemia, *n* (%)	31 (24.8)	16 (13.3)	0.025
Smoking, *n* (%)	90 (72)	10 (8.3)	0.0001
Anamnesis			
Family history of CAD, *n* (%)	76 (60.8)	42 (35)	0.014
History of myocardial infarction, *n* (%)	86 (68.8)	0	-
History of stroke, *n* (%)	11 (8.8)	0	-
Atherosclerosis of other pools, *n* (%)	21 (16.8)	0	-
No angina, *n* (%)	8 (6.4)	120 (100)	0.0001
Functional class I angina, *n* (%)	0	0	-
Functional class II angina, *n* (%)	51 (40.8)	0	-
Functional class III angina, *n* (%)	66 (52.8)	0	-
Chronic heart failure NYHA I functional class, *n* (%)	16 (12.8)	26 (21.7)	0.030
Chronic heart failure NYHA II functional class, *n* (%)	11 (8.8)	54 (45)	0.0002
Chronic heart failure NYHA III functional class, *n* (%)	7 (5.6)	40 (33.3)	0.003
Chronic heart failure NYHA IV functional class, *n* (%)	0	0	-
Atherosclerosis of the 1st coronary artery, *n* (%)	10 (8)	0	-
Atherosclerosis of the 2nd coronary artery, *n* (%)	6 (4.8)	0	-
Atherosclerosis of three or more coronary artery, *n* (%)	109 (87.2)	0	-
Ejection fraction, %	51.0 (44.13; 56.377)	53.2 (43.41; 58.03)	0.125
Treatment strategy/group of drugs (hospital period)			
Aspirin, *n* (%)	122 (97.6)	0	-
Clopidogrel, *n* (%)	21 (16.8)	0	-
Warfarin, *n* (%)	0	103 (85.8)	-
β-blockers, *n* (%)	122 (97.6)	111 (92.5)	0.312
Angiotensin-converting enzyme, *n* (%)	96 (76.8)	95 (79.2)	0.247
Statins, *n* (%)	125 (100)	98 (81.7)	0.033
Calcium channel Blocker, *n* (%)	96 (76.8)	90 (75)	0.151
Nitrates, *n* (%)	11 (8.8)	10 (8.3)	0.417
Diuretics, *n* (%)	105 (84)	110 (91.7)	0.062

Notes: Hereinafter: CAD, groups of patients with coronary artery disease and indications for direct myocardial revascularization by coronary artery bypass grafting, *p*, significance level.

**Table 2:** Indicators of quantitative assessment of epicardial, perivascular, abdominal fat depots in patients with cardiovascular diseases.

Parameters	CAD 1, (*n* = 125)	Heart Defects, (*n* = 120)	*p*
Thickness EAT LV, mm	3.53 (2.87; 4.36)	2.76 (2.42; 3.21)	*p* = 0.022
Thickness EAT RV, mm	4.58 (4.09; 6.17)	3.65 (3.12; 3.97)	*p* = 0.031
Thickness PVAT *p*/3 RCA, mm	4.55 (3.53; 5.76)	2.70 (2.41; 3.29)	*p* = 0.002
Thickness PVAT m/3 RCA, mm	4.67 (3.49; 6.13)	2.63 (2.24; 3.29)	*p* = 0.003
Thickness PVAT LCA, mm	3.64 (3.33; 4.46)	2.77 (2.48; 3.12)	*p* = 0.027
Thickness PVAT *p*/3 anterior descending artery, mm	4.31 (3.51; 4.87)	3.15 (2.56; 3.03)	*p* = 0.001
Thickness PVAT m/3 anterior descending artery, mm	3.71 (3.24; 4.92)	2.62 (2.16; 2.88)	*p* = 0.012
Thickness PVAT *p*/3 circumflex artery, mm	3.35 (3.12; 4.47)	2.74 (2.34; 2.93)	*p* = 0.037
Thickness PVAT m/3 circumflex artery, mm	3.67 (3.10; 4.99)	2.55 (2.27; 2.83)	*p* = 0.002
Area VAT, cm^2^	168.28 (149.21; 198.19)	136.24 (96.28; 142.13)	*p* = 0.031
Area SAT, cm^2^	264.51 (190.15; 311.46)	277.36 (197.12; 344.31)	*p* = 0.418

Notes. The data presented are the medians (25th quartiles and 75th quartiles). EAT, epicardial adipose tissue, LV, left ventricle, RV, right ventricle, PVAT, perivascular adipose tissue, *p*/3, proximal third, RCA, right coronary artery, m/3, middle third, LCA, left coronary artery, VAT, visceral adipose tissue, SAT, subcutaneous adipose tissue.

**Table 3:** Area under the ROC curve and 95% confidence interval for atherosclerotic coronary artery disease.

Parameters	AUC	p	95% Confidence Interval (CI)	
Thickness EAT LV, mm	0.871	*p* < 0.012	0.693	0.983
Thickness EAT RV, mm	0.611	*p* < 0.025	0.552	0.739
Thickness PVAT *p*/3 RCA, mm	0.706	*p* < 0.031	0.708	0.979
Thickness PVAT m/3 RCA, mm	0.649	*p* < 0.037	0.523	0.729
Thickness PVAT LCA, mm	0.793	*p* < 0.022	0.642	0.937
Thickness PVAT *p*/3 anterior descending artery, mm	0.832	*p* < 0.014	0.704	0.956
Thickness PVAT m/3 anterior descending artery, mm	0.626	*p* < 0.033	0.686	0.769
Thickness PVAT *p*/3 circumflex artery, mm	0.771	*p* < 0.019	0.631	0.847
Thickness PVAT m/3 circumflex artery, mm	0.611	*p* < 0.041	0.556	0.718
Area VAT, cm^2^	0.809	*p* < 0.026	0.694	0.963
Area SAT, cm^2^	0.598	*p* < 0.035	0.386	0.627

EAT, epicardial adipose tissue; LV, left ventricle; RV, right ventricle; PVAT, perivascular adipose tissue; *p*/3, proximal third, RCA, right coronary artery; m/3, middle third, LCA, left coronary artery; VAT, visceral adipose tissue; SAT, subcutaneous adipose tissue; AUG, area under the ROC curve.

**Table 4:** Gene expression IL-6 and IL-6 concentration in adipocyte cultures of various fat depots in patients with coronary artery disease and patients with aortic or mitral valve replacement.

Parameter	Subcutaneous Adipocytes		Epicardial Adipocytes		Perivascular Adipocytes		*p*	
	CAD	Heart Defects	CAD	Heart Defects	CAD	Heart Defects		
	1	2	3	4	5	6		
*IL6* expression, Delta Ct	0.037 (0.025; 0.051)	0.053 (0.034; 0.061)	0.077 (0.062; 0.081)	0.056 (0.049; 0.075)	0.048 (0.037; 0.057)	0.029 (0.021; 0.032)	0.012	P_1–3_ = 0.003 P_3–5_ = 0.011 P_2–6_ = 0.0001 P_4–6_ = 0.0001
	P_1–2_ = 0.124		P_3–4_ = 0.002		P_5–6_ = 0.0002			
IL-6, pg/mL	12.37 (9.12; 16.27)	13.92 (10.01; 16.26)	29.35 (25.18; 37.16)	21.55 (17.77; 23.34)	18.12 (15.61; 21.06)	11.64 (8.79; 14.17)	0.011	P_1–3_ = 0.0001 P_3–5_ = 0.0002 P_2–4_ = 0.022 P_4–6_ = 0.001
	P_1–2_ = 0.071		P_3–4_ = 0.022		P_5–6_ = 0.025			

The data presented are the medians (25th quartiles, 75th quartiles). *IL6*, interleukin-6 gene, IL-6, interleukin-6.

**Table 5:** Association of the level of expression and secretion of adipocytokines from local fat depots with an increase in morphometric parameters of adipose tissues in patients with CAD.

Parameters	Odds Ratio (OR)	95% Confidence Interval (CI)		*p*
ADIPOQ expression in EAT	0.47	0.39	0.53	*p* = 0.013
ADIPOQ expression in PVAT	0.63	0.55	0.71	*p* = 0.002
LEP expression in EAT	1.54	1.44	1.60	*p* = 0.022
IL6 expression in EAT	1.51	1.43	1.59	*p* = 0.002
IL6 expression in PVAT	1.41	1.35	1.50	*p* = 0.002
Adiponectin concentration in EAT	0.55	0.46	0.61	*p* = 0.001
Leptin concentration in EAT	2.52	2.46	2.60	*p* = 0.014
Leptin concentration in PVAT	2.34	2.25	2.40	*p* = 0.001
IL-6 concentration in EAT	1.49	1.40	1.58	*p* = 0.002
